# Replantation of Displaced Underlying Successor and Marsupialization of Radicular Cyst associated with a Primary Molar

**DOI:** 10.5005/jp-journals-10005-1287

**Published:** 2015-04-28

**Authors:** Gagandeep Lamba, GR Ravi

**Affiliations:** Reader, Department of Pedodontics, VSPM Dental College and Hospital, Nagpur, Maharashtra, India; Reader, Department of Pedodontics, Drs Sudha and Nageswara Rao Siddhartha Institute of Dental Sciences, Chinnoutpalli Andhra Pradesh, India

**Keywords:** Primary molar, Radicular cyst, Replantation.

## Abstract

Radicular cysts are by far the most common cystic lesions of the jaw. However, those arising from primary teeth are comparatively rare, comprising only 0.5 to 3.3%. The aim of this paper is to present clinical, radiographic and histopathological characteristics of radicular cyst associated with a primary mandibular molar causing unusual displacement of the permanent successor. Extraction of primary tooth along with extirpation of cyst was done under local anesthesia. The displaced premolar was also extracted and then replanted in the socket after proper alignment. Healing was uneventful and the space of missing primary molar was maintained by band and loop space maintainer. The relationship between intracanal medicaments and rapid growth of cyst, as mentioned in literature was observed in our case too. Thus, pulpotomy treated primary teeth should receive periodic postoperative radiographic examination and absence of clinical symptoms does not mean that a pulpotomy treated tooth is healthy.

**How to cite this article:** Lamba G, Ravi GR. Replantation of Displaced Underlying Successor and Marsupialization of Radicular Cyst associated with a Primary Molar. Int J Clin Pediatr Dent 2015;8(1):70-74.

## INTRODUCTION

Children are victims of many pathological lesions involving the jaw bones. These lesions can be neoplastic, developmental or Inflammatory in origin. Most common among the Inflammatory lesions is radicular cyst. These cysts are usually seen at the apices of the teeth with infected and necrotic pulp. It arises from epithelial residues in the periodontal ligament as a result of Inflammation, usually following death of pulp.^1^ These cysts are by far the most common cystic lesions in the jaws. Those arising from primary teeth are, however, rare, with a prevalence of 0.5 to 3.3%.^2,3^

Most radicular cysts develop slowly and do not become very large. They are usually asymptomatic unless there is a sign of acute Inflammatory exacerbation. They are often detected only during routine radiographic examination. If the cyst does become large, symptoms include swelling, mild sensitivity, tooth mobility and sometimes displacement of underlying tooth. Caries is the most frequent cause for radicular cyst in primary dentition. They may also form following traumatic injuries to primary teeth.^1^ Intracanal medicaments used for pulp therapy and distinctive intraepithelial inclusions found in cyst wall, may also provide a site for continuous antigenic stimulation.^4^

According to Formigli et al, radicular cysts represent potent bone destroying lesions of the maxillofacial region.^5^ Shear^3^ reported that resorption of primary teeth is rare in cases of radicular cyst although Neville^6^ et al stated it is common during cyst development. A definitive diagnosis should be based upon radiographic, clinical and histological pattern. The treatment for radicular cyst includes total enucleation if the cyst is small, marsupiali-zation for decompression of larger cysts or a combination of two techniques. Inflammatory cysts do not recur after adequate treatment.^1^

The aim of this report is to present clinical, radio-graphical and histological characteristics of radicular cyst associated with a primary molar following incomplete endodontic treatment, and the various conservative treatment modalities available to treat radicular cyst.

## CASE REPORT

**Fig. 1:**
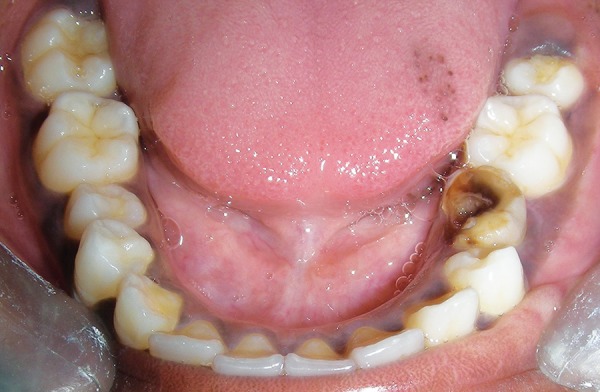
Clinical appearance of grossly carious 75

**Fig. 2:**
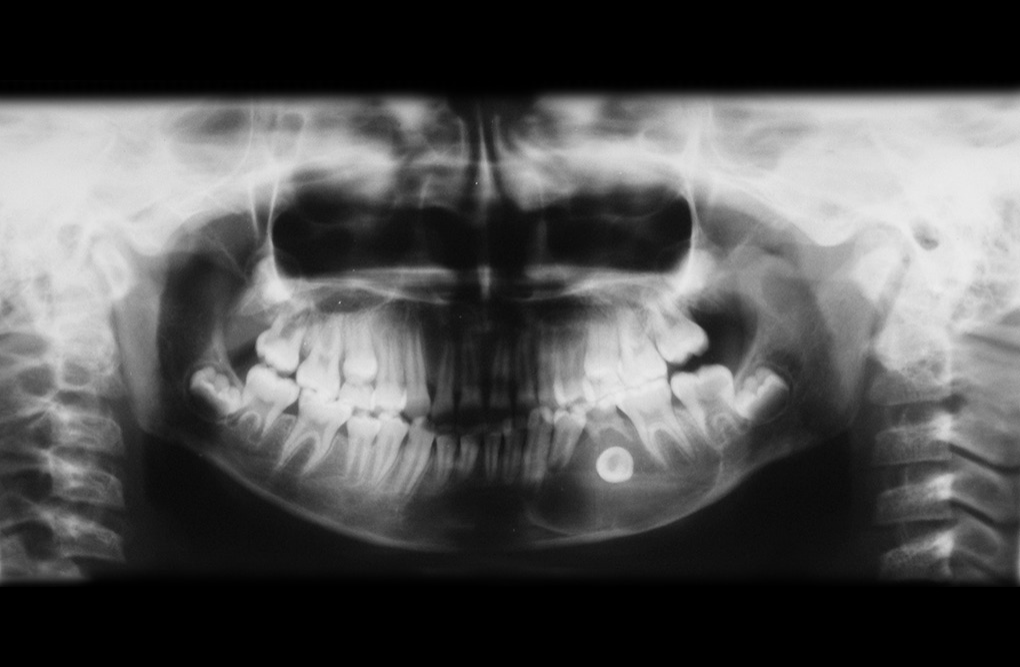
Panoramic radiograph at first examination showing unilocular radiolucent lesion, below the left second primary molar, displacing the successive permanent second premolar apically

**Fig. 3:**
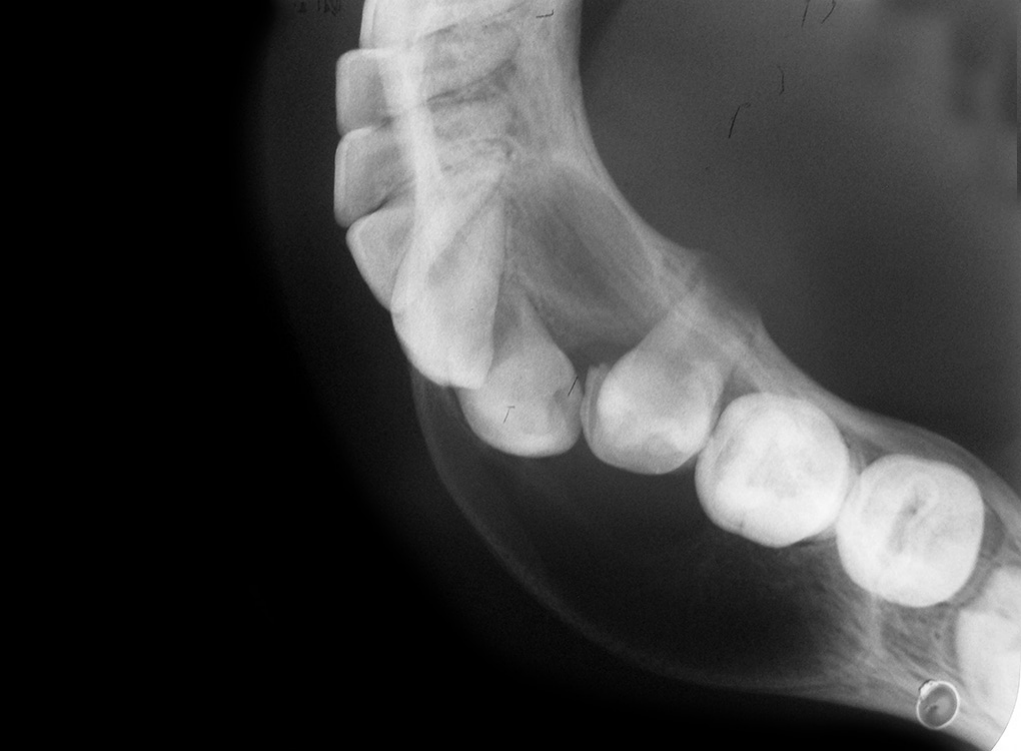
Occlusal radiograph showing buccal cortical plate expansion in relation to 75

An Indian girl, 12 years old, was referred to Department of Pedodontics and Preventive Dentistry of Dental College, for inspection of a painless swelling in the mandibular left primary molar region since 6 months. Past dental history revealed that she had undergone incomplete endodontic treatment with the same tooth a year ago. Her oral hygiene was good and was overall in good health. Extraoral examination revealed, a diffuse, bony nontender swelling on left side of jaw extending from corner of mouth to angle of mandible. While, intraoral examination revealed a grossly carious and mobile left primary second molar tooth (Fig. 1). Panaromic radiograph showed a round unilocular radiolucent lesion, 1.8 × 2 cm in diameter, below the left second primary molar (Fig. 2), displacing the successive permanent second premolar apically. A closer examination of the radiograph revealed that the border of the lesion appeared corticated and was smooth and well-defined. Occlusal radiograph revealed buccal cortical plate expansion extending from distal aspect of permanent mandibular left lateral incisor to mesial aspect of permanent mandibular left second molar and revealed an interesting finding that the successive premolar was displaced and aligned buccolingually in the cystic cavity (Fig. 3).

**Fig. 4:**
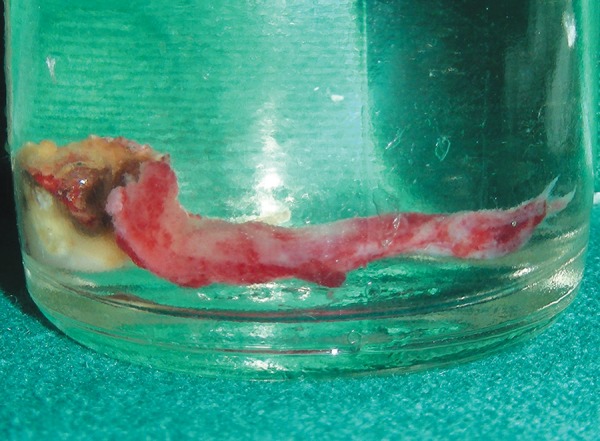
Cystic lining along with extracted 75

From history and clinical examination a provisional diagnosis of radicular cyst associated with them andibular left primary second molar was made which could be due to either secondary caries or the medicament used duri ng endodontic treatment. Differential diagnosis included periapical granuloma or dentigerous cyst.

**Fig. 5:**
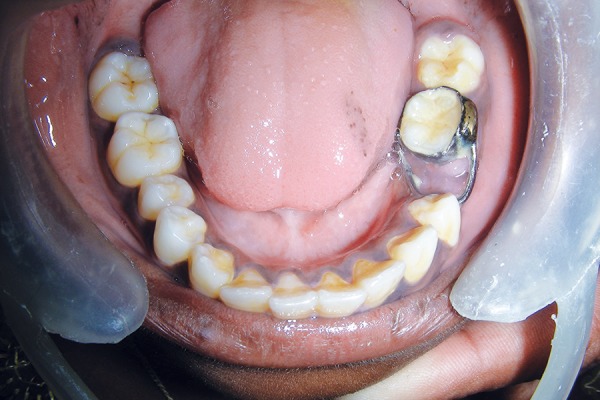
Intraoral view showing band and loop space maintainer

Primary left second molar was extracted under local anesthesia and cystic cavity was exposed. Cystic lining was removed as much as was accessible and sent for histopathological examination (Fig. 4). The successive permanent second premolar was also extracted because of its improper alignment. After thorough curettage of cystic lining, the premolar was placed back into the cystic bony cavity with proper alignment and a primary closure was attempted following debridement and hemostasis. During the entire procedure, care was taken to handle the periodontal surface as gently as possible with frequent immersion in normal saline solution. Surgical exploration confirmed that the cyst was not associated with permanent tooth. Postsurgical period was uneventful and short band and loop space maintainer was given after removal of the sutures to maintain the space (Fig. 5).

**Fig. 6:**
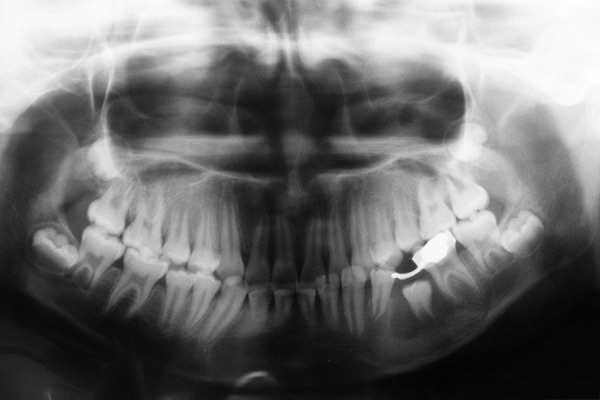
Orthopantomogram after 6 months follow-up showing short band loop space maintainer in relation to 75 and the successive permanent second premolar

**Fig. 7:**
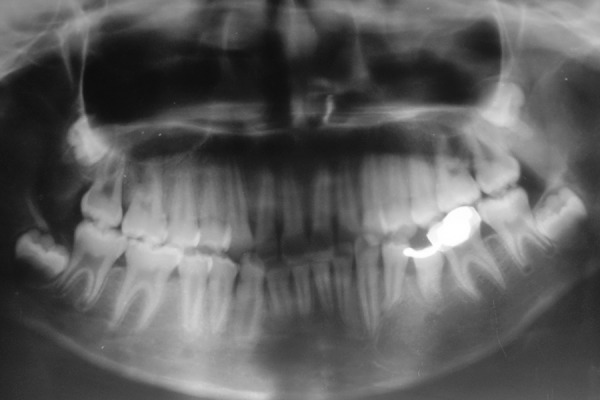
Postsurgery OPG taken after 18 months reveals significant bone formation

Histopathological features were consistent with provisional diagnosis of radicular cyst. Histological examination revealed the cystic cavity to be lined by a nonkeratinized stratified squamous epithelium with mixed Inflammatory infiltration. The patient returned once every 3 months for regular checkup. At 6 months follow-up, tooth had almost aligned vertically and was on the path of eruption (Fig. 6). At 18 months postsurgery, the OPG revealed good amount of bone formation (Fig. 7). The patient is on a regular follow-up.

## DISCUSSION

A radicular cyst is one which arises from the epithelial residues in the periodontal ligament as a result of Inflam-mation. The Inflammation usually follows the death of the dental pulp and cysts arising due to this are most commonly found at the apices of the involved tooth. Many radicular cyst s are sy mptomless and a re discovered when periapical radiographs are taken of teeth with nonvital pulps.^7^ In our case also, the large radicular cyst was an incidental finding on panoramic radiograph.

According to Shear and Lustman, radicular cysts are considered to be rare in primary dentition, accounting to less than 1% of all cases,^3^ while Lustman in an extensive review from 1898 to 1985, found only 28 cases to which they added 23 cases.^2^ Nagata et al in their review reported 112 cases till 2004. Since then, there have been few individual case reports.^8^ However, according to Mass et al, some factors characteristic of primary dentition can lead to an underestimation of real frequency of these lesions. Periapical radiolucencies relating to primary teeth tend to be neglected and mostly resolve after removal of the tooth.^9^ In addition, formation of sinus or fstula causes less severe symptoms as compared to permanent teeth and these radicular lesions may remain untreated. Other factors, such as diagnostic errors, extraction of primary tooth without referral for pathological examination and regression of the lesion due to endodontic treatment may also influence the reported frequency.^1^

There are several differences between radicular cysts originating from primary teeth and those originating from permanent teeth. In primary teeth mandibular molars are more commonly involved while in permanent teeth, maxillary anteriors are more commonly involved. The difference in site distribution may be explained by different etiologic factors. In primary dentition, caries is the most frequent etiologic factor and mandibular molars are more frequently affected teeth. In permanent maxillary incisors, the high frequency of radicular cyst results from trauma, caries and old silicate restorations.^3^ In primary molars, cyst is located in interradicular area and around the roots, whereas in permanent incisors, it is usually located at the apex. This can be explained by the short and sometimes partially resorbed roots of primary molars and also the presence of accessory canals. Thus, the term periradicular cyst in primary molars is more appropriate than radicular cyst.^7^

Periapical radiolucencies relating to primary teeth tend to be mi sdiag nosed as a periapical gra nu loma of pri mary teeth or dentigerous cyst from permanent successors. Radiographically, a dentigerous cyst is characterized by a unilocular radiolucent area that is associated with crown of an unerupted permanent tooth.^4^ But, the histological similarity between the lining of these two cysts points out the difficulty of differentiating between these two cysts on the basis of their histological features alone.^10^

On the other hand, periapical granulomas are radiographically indistinguishable from radicular cysts. Radicular cyst development is the next stage following formation of periapical granuloma.

Furthermore, larger the lesion, the greater the probability it will be a radicular cyst. Nearly all lesions > 2 cm in diameter are diagnosed as cysts.^4^ The growth rate estimated by Livingston^11^ and Hill^12^ were 5 and 4 mm annually, respectively. Grundy, Adkins and Savage reported a series of cases of radicular cysts associated with deciduous teeth that had been treated endodontically with materials containing formocresol, which in combination with tissue proteins, is antigenic and has shown to elicit a humoral and cell mediated response.^13^ This could be the probable reason for rapid growth of the cyst, displacing the underlying premolar buccolingually. In our case, the lesion was approximately 2 cm and during the surgical procedure, it was noticed that the lining was not attached to the crown of the underlying tooth, ruling out the diagnosis of dentigerous cyst. Also, as suggested by patient’s history relating to previous treatment, there could be a probability of use of formocresol. Thus, all these factors along with histological confirmation, together contributed to reach the final diagnosis of radicular cyst.

The most common clinical and radiographic features of radicular cyst in primary molars include the following:

1. Mandibular buccal cortical plate expansion
2. Well-defined unilocular radiolucency
3. Thin reactive cortex
4. Displacement of succedaneous tooth
5. Misleading preoperative diagnosis^2^

Our case was diagnosed as a radicular cyst in the primary dentition for following reasons: presence of large and painless radiolucent lesion in relation to roots of a nonvital primary tooth, size larger than 2 cm, no clinical and radiographic involvement with successive permanent tooth, displacement of the underlying successive tooth, histological confirmation of cystic epithelial lining, and surgical confirmation that lesion was not associated with the successive permanent tooth.

Treatment choices include extraction of the primary tooth and preservation of underlying permanent tooth. If there is no threat of damage to underlying permanent tooth bud, complete curettage can be performed. If permanent tooth is severely damaged, complete enuclea-tion of the cyst to include the permanent tooth bud has been recommended.^14^

In the present case, curettage was adopted, along with removal of the underlying premolar, as it was not properly aligned. Extraoral time in this treatment was around 6 to 8 minutes. According to Kratchman extraoral time should not exceed 10 minutes.^15^ The premolar was again replanted into the cystic cavity and the cystic cavity was sutured. Following the removal of sutures after 7 days a short band and loop was cemented to maintain the space till the eruption of the permanent premolar. The patient is under regular follow-up.

## CONCLUSION

On the basis of our case, we conclude that:

1. Practitioners should investigate cases of delayed exfoliation and expansion of cortical plate in vicinity of primary molars.
2. Pulpal therapeutic agents may causeantigenic stimuli but it does not imply prohibition of use of medicaments.
3. Regular clinical and radiographic follow-up of endo-dontically treated primary teeth should be done. Absence of clinical symptoms does not imply that the tooth is healthy.
4. Bone grafts for large lesions are not required in children because of high propensity for bone regeneration.
